# Theranostic extracellular vesicles: a concise review of current imaging technologies and labeling strategies

**DOI:** 10.20517/evcna.2023.01

**Published:** 2023-03-30

**Authors:** Safiya Aafreen, Jonathan Feng, Wenshen Wang, Guanshu Liu

**Affiliations:** 1 Department of Biomedical Engineering, Johns Hopkins University, Baltimore, MD 21218, USA.; 2 F.M. Kirby Research Center for Functional Brain Imaging, Kennedy Krieger Institute, Baltimore, MD 21205, USA.; 3 Russell H. Morgan Department of Radiology and Radiological Sciences, The Johns Hopkins University School of Medicine, Baltimore, MD 21205, USA.

**Keywords:** Extracellular vesicles, exosomes, theranostics, molecular imaging, cell-free regenerative medicine

## Abstract

Extracellular vesicles (EVs), or exosomes, are naturally occurring nano- and micro-sized membrane vesicles playing an essential role in cell-to-cell communication. There is a recent increasing interest in harnessing the therapeutic potential of these natural nanoparticles to develop cell-free regenerative medicine and manufacture highly biocompatible and targeted drug and gene delivery vectors, amongst other applications. In the context of developing novel and effective EV-based therapy, imaging tools are of paramount importance as they can be used to not only elucidate the underlying mechanisms but also provide the basis for optimization and clinical translation. In this review, recent efforts and knowledge advances on EV-based therapies have been briefly introduced, followed by an outline of currently available labeling strategies by which EVs can be conjugated with various imaging agents and/or therapeutic drugs and genes. A comprehensive review of prevailing EV imaging technologies is then presented along with examples and applications, with emphasis on imaging probes and agents, corresponding labeling methods, and the pros and cons of each imaging modality. Finally, the potential of theranostic EVs as a powerful new weapon in the arsenal of regenerative medicine and nanomedicine is summarized and envisioned.

## INTRODUCTION

**Figure 1.**
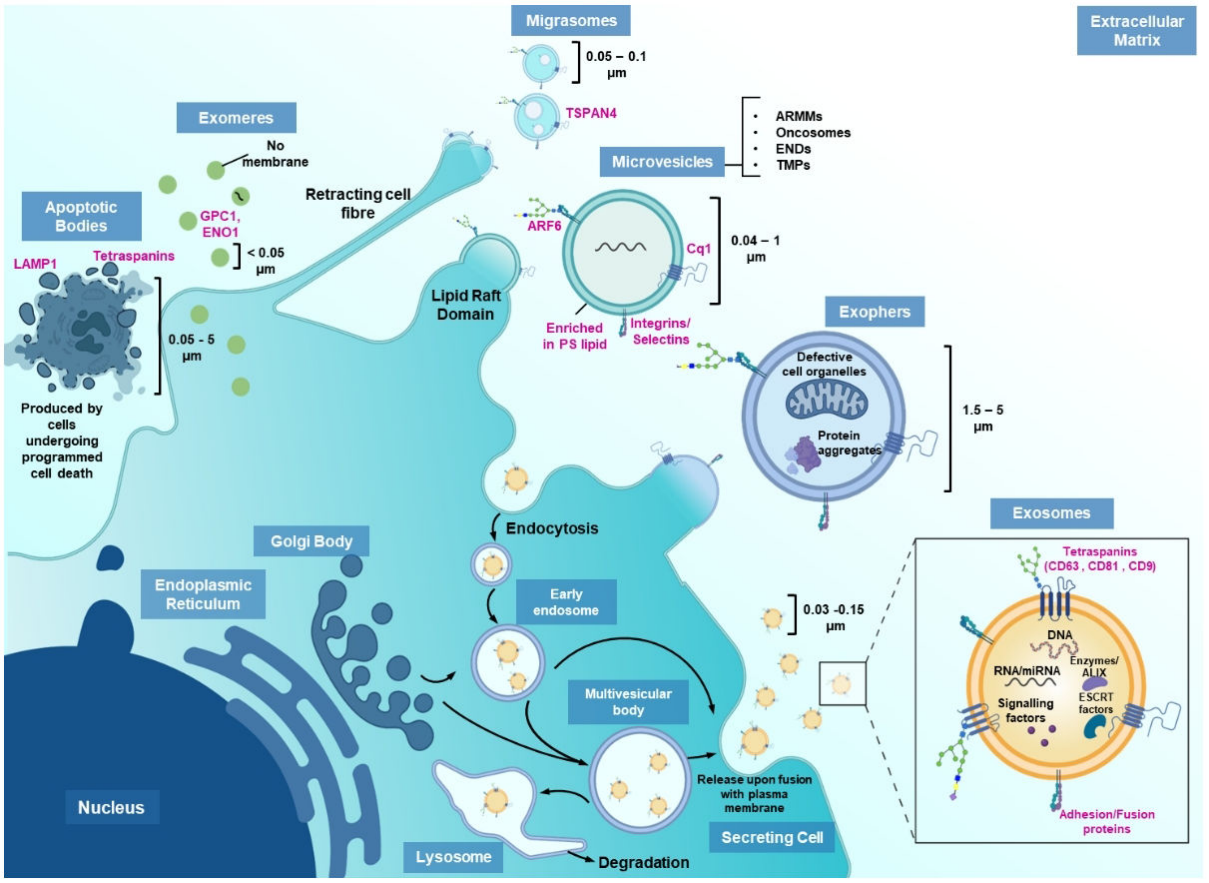
Heterogenous EV populations secreted by cells. The illustration depicts the general characteristics of various subpopulations of EVs, namely exosomes, microvesicles, apoptotic bodies, exophers and migrasomes. EV-identifying markers have been labeled in pink. The figure was created using assets from Biorender.com

In 1836, Darwin, in his theory of Pangenesis, postulated the existence of particles shed by all cells in an organism that can circulate in the body and facilitate genetic transfer^[1]^. Like evolution, the hypothesis was first ridiculed before the scientific community realized the veracity of the statement and are now expediating research into the understanding fundamental biology, cell communication, and disease progression, which have allowed us to design cell-free therapeutics using these naturally occurring nano- and micro-sized vesicles, which we now collectively call extracellular vesicles (EVs). Based on recent literature, EVs are defined as a heterogenous group of membranous carriers secreted by all cells, from prokaryotes to eukaryotes^[2,3]^. Historically, by their size and biogenesis, EVs are categorized as: (A) exosomes (endosomal origin, 30-150 nm) that are secreted under homeostatic and stressed conditions via the endosomal sorting complex dependent or independent pathway. They contain various membrane proteins, cytosolic proteins, dsDNAs, RNAs/siRNAs/miRNAs, lipids and signaling factors that aid in intercellular communication, including components for repair or cell death resistance^[4,5]^; (B) microvesicles (membrane blebbing, 40-1,000 nm) that include the newly discovered subpopulations of ARMMs (arrestin domain-containing protein 1 mediated microvesicles), tumor-derived oncosomes, neutrophil ENDs (elongated neutrophil-derived structures) and TMPs (T cell microvilli particles). They mainly encompass cytosolic contents, including proteins and RNA strands^[6,7]^; (C) Apoptotic bodies (cytosolic skeleton, 0.05-5 μm) that act as suicide notes to the surrounding cells and contain fragmented nuclear particles and proteins from karyorrhexis and cell collapse; (D) Exophers (membrane blebbing, 1.5-5 μm) that are evidenced to be released by the soma and cardiomyocytes and contain damaged organelles such as mitochondria and lysosomes along with cytosolic protein aggregates and are produced by physiologically normal cells. These may spread pathological proteins if not eliminated by the immune system^[8]^; (E) Migrasomes (retracting fibers of migrating cells, 50-100 nm), which are released from the tip of retraction fibers, are pomegranate-like vesicles left behind by migratory cells and consist of cytosolic growth factors, mRNAs, proteins and fragments of damaged mitochondria^[9]^; (F) Exomeres (< 50 nm), which are non-membranous nanoparticles, are enriched in proteins involved in metabolism and may modulate glycosylation in recipient cells [Figure 1]. Based on these factors, exosomes are desirable for theranostic applications as they are secreted with components from the Golgi and endosomal system, which would be more specific in function. However, given the fact that EVs are often heterogeneous in the small size range and purification based on biogenetic origin is formidably difficult, if not totally impossible, a new classification system has been recently proposed to categorize EVs simply by their size into “small EVs” (100 nm or < 200 nm) and “medium/large EVs”(> 200 nm)^[4,10]^. For most applications, exosome- like EVs are preferred and small EVs are, thereby, utilized as most EVs that would have the predisposition to be cytotoxic would fall under the umbrella of medium/large EVs. Small EVs also confer the ability to provide a larger number of agents to target cell populations.

EVs have been intensively investigated as a new type of therapeutics falling in the category of biologies. For instance, while the underlying mechanism is not fully understood, mounting evidence has shown that EVs exert protective and/or reparative effects through cytoprotection, stimulation of angiogenesis, induction of antifibrotic cardiac fibroblasts, and modulation of polarization of M1/M2 macrophages for infiltration of the infarcted region^[11,12]^. Compared to their parental stem cells (SCs), human SC-EVs being cell-free, are a priori safer and more effective regenerative medicine approach for treating many diseases^[13-15]^. Their smaller size reduces the chances of thrombosis and allows for easy transport and *in vivo* administration of soluble therapeutics with the potential for long-term storage. As a primary paracrine executor of stem cells, SC-EVs circumvent the restrictions posited by SC therapy, such as immunogenicity, tumorigenesis, and unwanted cell differentiation. However, to accomplish satisfactory outcomes, efficient delivery of SC-EVs to the injured tissues needs to be established first. Currently, clinically preferred systemic administration has poor efficacy due to the non-specific uptake of intravenously infused EVs by the liver and spleen^[16-19]^. New engineered EVs with higher disease-targeting ability are proposed, yet vigorous testing and validation are still lacking. Imaging approaches that can timely monitor and quantify the delivery of EVs is highly desired, and integration of imaging into therapeutics allows the development of theranostic EV systems^[20-22]^. Moreover, such imaging tools are also of paramount importance for later translational development and clinical applications of EV therapy.

In this review, we will first introduce recent efforts and knowledge advances on EV-based therapies and outline currently available labeling strategies by which EVs can be conjugated with various imaging agents and/or therapeutic drugs and genes. A lack of information regarding EV fate *in vivo* still hinders their translation into clinical settings. Non-invasive imaging of EVs *in vivo* can be paramount to facilitate EV-based therapeutic development, transition to clinical trials and even personalized regimens by monitoring biodistribution and quantifying localized concentrations. To this intent, a comprehensive review of prevailing EV non-invasive imaging technologies is presented along with examples and applications, with emphasis on imaging probes and agents, corresponding labeling methods, and the pros and cons of each imaging modality. Finally, we will provide a summary and outlook on the potential of theranostic EVs as a powerful new weapon in the arsenal of regenerative medicine and nanomedicine.

## EVS AS THERAPEUTICS

To date, EVs have been found to be connected to almost all human diseases, including COVID-19^[23]^, cardiovascular diseases^[12,24]^, neurodegeneration^[25,26]^, cancer^[27]^, and liver diseases^[28]^, just to name a few. A recent study showed that EVs isolated from the plasma of COVID-19 ICU patients contained an elevated amount of D-dimer values, tenascin-C (TNC) and fibrinogen-β (FGB) relative to that of healthy controls i.e., volunteers who had not contracted the disease. This was shown to contribute to the promotion of pro-inflammatory cytokines via the Nuclear factor-κB pathway at organs not in proximity to the site of infection and had led to severe tissue damage in patients. In multiple sclerosis, EVs can cross the blood-brain barrier and pass brain antigens along to peripheral immune cells^[29]^. B cells also secrete EVs consisting of substantial amounts of accessory molecules, such as B7, ICAM-1, and LFA-3 and functional MHC class II molecules associated with peptides which sparked a powerful antigen-specific T helper response^[30]^. Increased evidence also shows that cancer cells secrete EVs with apoptosis inducing ligands such as FasL and galectin 9 to abrogate immune response^[31]^. Macrophage immunosuppressive polarization has also been exhibited upon engulfment of bladder cancer- derived EVs, specifically by down-regulation of PTEN and activation of AKT/STAT3/6 signaling using miR-1231-sp microRNAs^[32]^. Red blood cell EVs can be a safe versatile delivery system for therapeutic RNAs with no inherent DNA content and have been shown to be loaded with miR-125b antisense oligonucleotides to suppress breast tumors^[33]^.

The latest version of the EV content database, Exocarta (Version 6, http://www.exocarta.org), states that at least 9,769 proteins, 3,408 mRNAs, 1,116 lipids and 2,838 microRNAs have been identified in EVs originating from different cells and organisms and as a caveat, an immense diversity in effects exerted on target cells is observed^[34]^. Some instances of the effects of this mode of inter-cellular communication include the onset and progression of preeclampsia in birthing women conducted by EVs enriched in S100 calcium-binding protein B (S100b), serpin peptidase inhibitor (PAI)-1, porphyria cutanea tarda (PCT), natriuretic peptide B (BNP), TGF-β, VEGFR1, and placental growth factor^[35]^. For example, in the context of amyotrophic lateral sclerosis (ALS), an incurable neurodegenerative disease, new evidence suggests that EVs package and transport key proteins involved in the progression of including SOD1, TDP-43, dipeptide-repeat proteins (DPRs), and fused in sarcoma (FUS) between glial cells^[36]^. Cancer progression is highly dependent on the transfer of soluble factors for the proliferation of oncological cells. In gastric cancers, exosomal CD97 tetraspanin proteins were found to promote cell proliferation through the MAPK signaling pathway^[37]^. Additionally, tumor-derived EVs can alter the capacity of malignant cells to invade. Nasopharyngeal carcinoma-derived EVs can possess epithelial to mesenchymal transition (EMT)-inducing signals, including TGF-β, Hypoxia-Inducible Factor 1 alpha (HIF1α) and Matrix Metalloproteinases (MMPs)^[38]^. These studies imply the potential clinical values of EVs as diagnostic markers.

On the other hand, tremendous efforts have been made to explore EVs as therapeutics. Many native stem cell-derived EVs have been found to exert protective effects on injured tissue and defend them from disease-induced harm. Mesenchymal stem cell (MSC)-derived EVs confer the advantages of having lower immunogenicity and tumorigenicity over their parental cells and may prove to be novel therapeutics^[39,40]^. EVs can also be used as drug carriers to deliver therapeutic small-molecule drugs, including CRISPR-Cas9, siRNA and proteins, with the vesicle being engineered for retention in targeted tissues^[41]^. Benefits from an EV-based delivery system include their inherent homing ability, which permits specific cell targeting and delivery of bioactive agents over a long distance *in vivo*, even across the blood-brain barrier, which is a prime hindrance to conventional therapeutics. Their size is also ideal for phagocytosis and lysosomal evasion and membrane fusion^[42,43]^. As the characteristics of the cell of origin are transferred to their secreted EVs, we can utilize non-immunogenic models such as those derived from stem cells to ultimately fabricate off-the-shelf products and use inherent EVs in circulation as unique fingerprints for extracting cell state information. Moreover, EVs possess a hydrophilic core which precludes the requirement of chemical modification of therapeutics for improved pharmacokinetics. Current bottlenecks of EV therapeutics being routinely used clinically include a lack of standardized isolation and purification methods. Five isolation methods have been developed: (1) ultracentrifugation-based isolation techniques; (2) immunoaffinity capture-based techniques; (3) size-based isolation techniques; (4) microfluidics-based isolation techniques; and (5) precipitation. Ultracentrifugation is the most commonly used by researchers. An ideal isolation technique would be selective, convenient, economical, reproducible, high-yield, time-saving, and high-throughput. Based on the MISEV2018 criteria, none of the isolation methods meets this standard and technological advances need to be made to establish standardized isolation^[44]^. A second limitation is the limited space available to load therapeutics in the lumen of EVs. GMP-grade EV production also needs to be developed that ensures a sterile, sufficient therapeutic payload and batch-to-batch reproducible method. Thereby, research pertaining to *in vivo* biodistribution of EVs is paramount to combat these restraints.

There are 20 companies globally involved in EV -based therapeutics, with some currently undergoing clinical trials targeting pancreatic cancer and neuromuscular diseases^[45]^. EVs can be utilized for immune modulation in alleviating illnesses with no current cures, such as sepsis which has a high mortality rate, especially in underdeveloped regions, and is a syndrome associated with severe infection^[46,47]^. Investigations into the transcriptional changes and blockade of the nuclear factor-κB pathway have resulted in the development of EVs loaded with a super-repressor IκB kinase that is resistant to degradation and exerts its effect by blocking NF-κB’s nuclear translocation. This is observed even in the presence of pathogens that could arise in a pro-inflammatory environment, thereby inhibiting the expression of the pathway’s target genes^[47]^. Current challenges to utilizing EVs as a drug-delivery platform include technical limitations involving scalability, yield after isolation and commercialization expenses. As EVs are a relatively new therapeutic model, there are no standard guidelines available for their optimum storage and increasing specific yield. Electron microscopy and particle size distribution analysis methods are widely used for characterization in the field, which does not suffice as they can be confused with newly discovered particles like exomeres, and thereby additional reliable methods for *in vitro* and *in vivo* quality control and characterization methods are required^[48]^.

## THE ART OF OBSERVATION: NON-INVASIVE IMAGING METHODS FOR EV TRACKING *IN VIVO*

**Figure 2.**
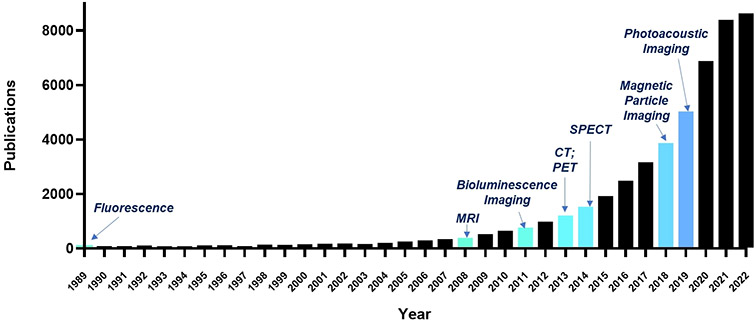
Milestones in EV imaging. Keywords including “exosome” or “extracellular vesicle”, and imaging modalities were referenced in PubMed (https://pubmed.ncbi.nlm.nih.gov/) to obtain articles that employed a particular imaging technology for the first time to observe EVs. The bar graph represents the total number of publications on EV research per year.

The optimized efficacy and clinical translation of EV-based therapeutics will be greatly benefited from full comprehension of EV biodistribution, pharmacokinetics and pharmacodynamics, for which *in vivo* tracking is a crucial tool. Imaging methods that allow EV monitoring *in vivo* offer great advantages over the traditional ex vivo methods, which require sacrifice of the animal and only can provide a snapshot at a fixed time point. However, imaging EVs with acceptable sensitivity and specificity is not an easily accomplished task due to their small particle size and extremely low quantity yield (i.e., 10^7^ particles/mL of culture cell medium). Over the last three decades, tremendous efforts have been made to explore molecular imaging modalities for EV imaging [Figure 2]. These methods include magnetic resonance imaging (MRI), optical imaging, nuclear imaging, magnetic particle imaging (MPI), photoacoustic imaging (PAI), and computed tomography (CT)^[49-51]^. In this section, labeling strategies by which imaging agents are either chemically or physically attached to EVs will be discussed. Then a brief walkthrough of each imaging modality and associated imaging agents, along with examples, will be provided, followed by a discussion of the advantages and disadvantages of each molecular imaging technique.

### Labeling strategies

The strategies for EV labeling can be categorized into two types, direct and indirect labeling^[52]^, by whether the EVs or their parental cells are used in the labeling step. In an indirect EV labeling study, parent cells are labeled with imaging agents first, and then the secreted EVs are subsequently collected. A portion of the collected EVs contains the imaging agents passed on from parental cells. Loading of imaging agents can be passive or active. Passive loading employs the same techniques that have been developed for cell labeling and imaging. Active loading procedures involve the manipulation of the EVs biogenesis pathway in order to incorporate bioagents. For example, the parent cell could be genetically modified to overexpress the therapeutic or nucleic acid material internally, which would lead to its subsequent shuttling into the vesicle^[53]^. However, genetic manipulations of parent cells could pose hurdles to clinical approval, as it raises concerns about malignant differentiation. Some companies such as System Biosciences have achieved docking of bioactive agents by specifically targeting endosomal proteins with peptide sequences for fusion. However, these therapeutics have restricted mobility and biological activity^[54,55]^. Genetic engineering of EV-releasing cells usually involves the expression of luciferase introduced through plasmid transfection. Alternately CRISPR/Cas9 systems can target EV membrane marker proteins such as CD63 to express GFP^[56]^.

While it has been demonstrated in many studies, this strategy often suffers from low and unstable labeling efficiency. Only a small subset of EVs can be obtained with labeling. Moreover, genetic modification may also change certain properties in the cell, which are then reflected in the resulting EVs. The transfection may also not be ubiquitous, which leads to an overall decrease in the yield of imaging probes^[57,58]^. In contrast, the direct labeling strategy works directly on EVs, providing repeatable and reproducible labeling efficiency. In this section, we will focus on the direct labeling strategy and interested readers can be referred to a Arifin’s review paper^[52]^.

**Figure 3.**
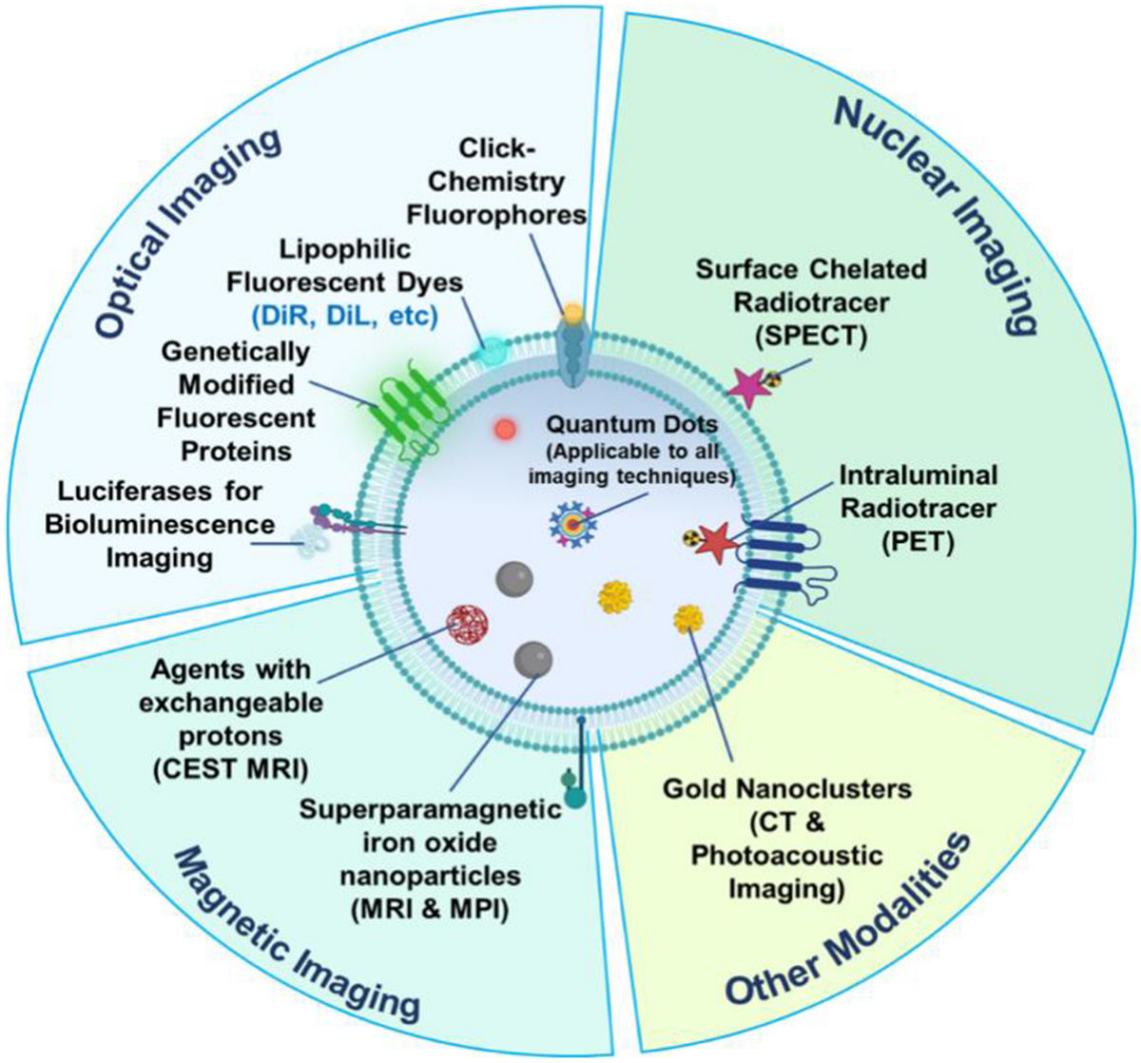
Imaging labels used in different imaging modalities^[59]^. Image and subsequent images were created using assets from Biorender.com.

Strategies to label EVs directly can be further classified into physical (incubation, sonication, extrusion, and electroporation) and chemical (EV surface chemo-modification). Many of these methods are the same as those for loading drugs or genes to synthetic nanoparticles. In this section, imaging probes are referred to as fluorescent and bioluminescent proteins, radioisotopes, and various nanoparticles such as aggregation-induced emission luminogens, superparamagnetic iron oxide nanoparticles (SPIONs) and quantum dots, which will be discussed in detail in the next section [Figure 3].

#### Direct incubation

EVs consist of a lipid membrane that can readily be labeled by the simple incubation of near-infrared wavelength lipophilic dyes such as DiR, Cy7, PKH, DiD, and membrane permeant dyes such as Calcein AM, which can detect the presence of intact vesicles due to fluorescence being dependent on the presence of internal esterases^[60]^. One drawback of this labeling method is that the labeled fluorescent dyes can be detected in tissues even after degradation or internalization of EVs by target cells. Lipid labeling is not specific for intact EVs and can lead to false-positive detection occurring from the diffusion to cell membranes or cellular debris. Another drawback is that, after entering blood circulation and eventually extravasating in tissues, the lipophilic dyes may accelerate the aggregation of EVs. A study revealed that the dye-based labeling affected EV organotropism and resulted in varied biodistribution^[61]^. Additionally, purification of the probe involves multiple washing culminates in significant EV damage. When combined with conventional methods of EV isolation, this strategy leads to poor yield and labeling of other EV classes.

#### Electroporation

This labeling technique involves the generation of temporary micropores in the phospholipid membrane of the vesicle upon application of an electric field for increased permeability of labeling agents such as SPIONs. Electroporation is a well-established technology in cell labeling for cell engineering and imaging, and encapsulating nucleotides into liposomes for gene therapy. However, this strategy decreases membrane integrity, requires removal of free label, and causes aggregation of loaded particles as well, which reduces the subsequent yield and can lead to false detection in off-target organs such as the liver and kidneys due to the amount of loaded agents leaked from the vesicle *in vivo*^[18,62]^.

#### Sonication

Similar to electroporation, sonication involves the application of an external mechanical shear force for diminishing the robustness of EV membranes and generating minute ruptures which permits contrast agent loading. The highest bottleneck to this method is the duration required for reversion of the damaged membrane structure, which could take a minimum of an hour, resulting in insufficient loading efficiencies^[63]^.

#### Extrusion

Extrusion is a physical procedure wherein EVs and cargo are passed repeatedly through an extruder with controlled nanopore size membranes to produce membrane recombination. This process results in a high loading efficiency, albeit at the expense of their immune-privileged status upon the recombination of EV surface structures, making them susceptible to immune cells like mononuclear phagocytes^[64]^. Moreover, the loss of intrinsic cargo is unpreventable.

#### Surface modification

Surface modification EVs can be conducted via covalent binding involving crosslinking reactions, namely azide-alkyne cycloaddition, colloquially known as click-reactions^[65]^. This involves the formation of a stable triazole bond. Receptor -ligand binding methods, including probes such as fluorescently tagged tetraspanin specific antibodies and aptamers, are also investigated^[66]^. The negative charge density of the EV membrane can also be exploited to utilize a multivalent electrostatic approach based on interactions with highly cationic species. This strategy, although efficient, tends to increase the overall size of the particle and, therefore, alters pharmacokinetics and bio-functionality^[65,67]^. Inconsistent findings have been noticed between studies that used the same cell line-derived EVs. In Peinado *et al.*’s study, B16F10- derived EVs labeled were fluorescently labeled and had accumulated in lung sections of mice at all time points after IV administration, whereas results reported in Faruqu *et al.* showed that B16F10 - derived EVs radiolabeled with ^111^In did not localize to the lungs in appreciable amounts at any time point, with the majority of detected particles in the liver and spleen^[68,69]^.

### Imaging modalities: seeing is believing

#### Optical imaging

Optical imaging was the first non-invasive imaging modality to visualize EVs *in vivo*. It is a ubiquitous technique in the molecular and cellular biology field with the advantages of high-throughput efficiency and low cost. It consists of two subtypes: fluorescence imaging (FLI) and bioluminescent imaging (BLI).

##### Fluorescence imaging

**Figure 4.**
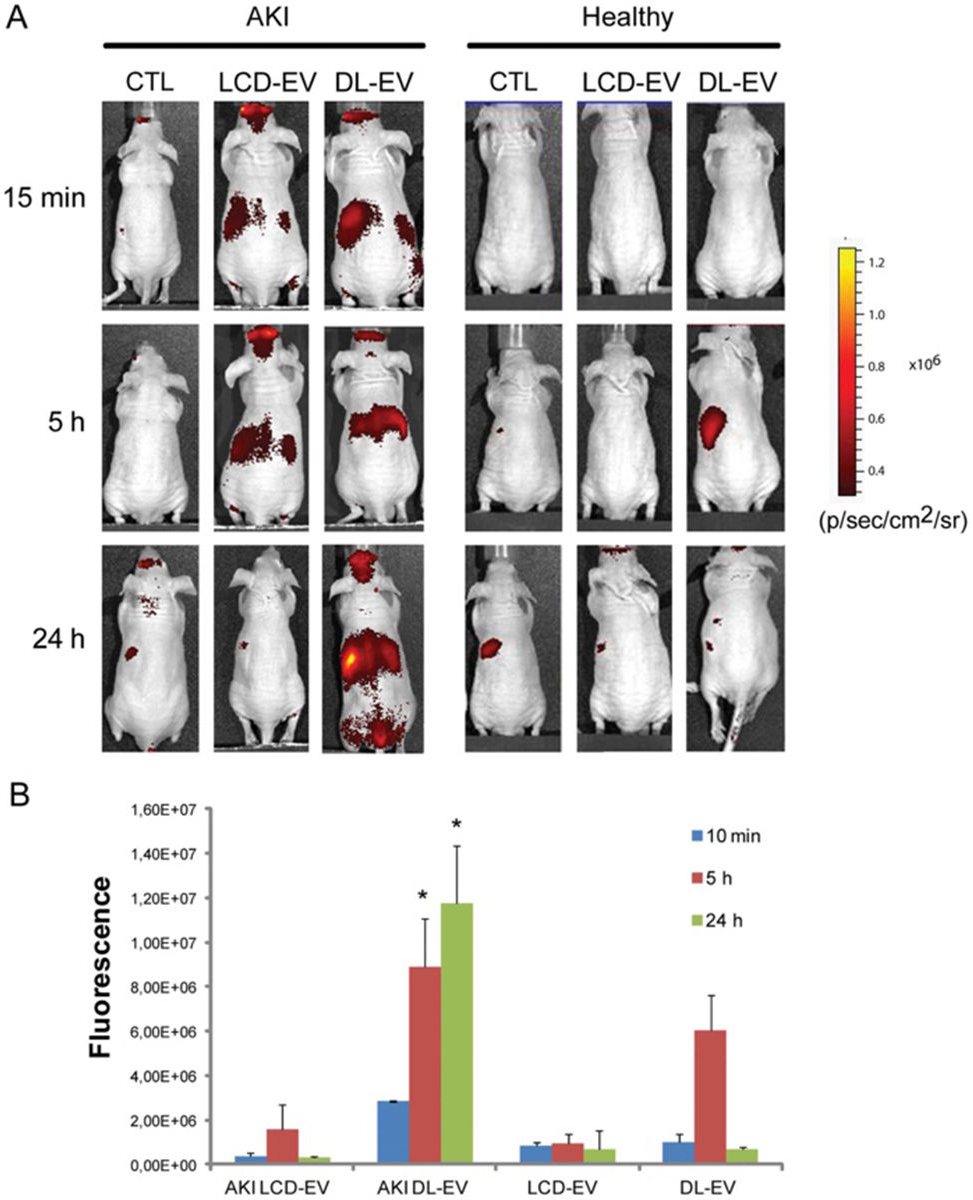
*In vivo* cell-derived extracellular vesicle (EV) bio-distribution in abdominal area by optical imaging (OI). (A) Representative OI images, acquired the supine position following the induction of acute kidney injury (AKI) in mice and in healthy mice treated intravenously with 200 μg of labeled cell-derived EVs (LCD-EVs) or directly labeled EVs (DL-EVs) or with an equal volume of phosphate-buffered saline (PBS) (CTL). (B) Quantification of fluorescence intensity in regions-of-interest (ROI) draw free hand in the abdominal area, expressed as the average radiance ± standard deviation (SD). Sixteen AKI mice were treated with LCD-EVs, 11 AKI mice were treated with (DL-EVs); healthy mice received the same amount of LCD- and of DL-EVs (*n* = 12 for LCD-EVs and *n* = 6 for DL-EVs). ANOVA with Newman-Keuls multicomparison test was performed. *P < 0.01 AKI DL-EV *vs.* all the other groups. LCD-EV, labeled EVs produced by donor cells; DL-EV, directly labeled EVs. Reprinted with permission from^[16]^.

To date, optical imaging remains the most widely used imaging technology to study the biodistribution of EVs in preclinical animal models [Figure 4] as well as a validation method for other imaging modalities, both *in vivo* and ex vivo.

The prevalence of fluorescent dyes for EV labeling, tracking, and imaging is the highest relative to other imaging modalities. Carbocyanine dyes, PKH dyes, and Azadibenzylcyclooctyne (ADIBO) dyes are just a few of the commercial dyes developed for this purpose. Carbocyanine dyes belong to the class of lipophilic dyes, which can spontaneously embed into EV membranes upon which they diffuse throughout the phospholipid bilayer and mark the entire vesicular structure. DiR, DiD, Cy5 and DiL are commonly used carbocyanine dyes with an emission wavelength falling in the near-infrared (IR) range that aids in image penetration depth. For example, Mirzaaghasi *et al.* studied the biodistribution and pharmacokinetics of DiR-labeled, HEK293T cell-derived EVs in a mouse sepsis model^[70]^. Results of the experiment highlight the sepsis-specific accumulation of EVs with a substantial amount inhabiting the lung after intravenous injection. No such targeting was exhibited by PEG-liposomes, which underscores EVs’ potential as injury-targeted therapy. PKH dye molecules, although belonging to the same category of dyes as carbonyl cyanine dyes, differ in structure as they possess a long aliphatic tail that can integrate into the lipid bilayer with an exposed hydrophobic fluorophore. Alternatively, fluorescence dyes can be conjugated to EVs via covalent bonding, either directly to membrane motifs on EVs or by the manipulation of the natural biosynthetic pathway of glycosylation. For example, Santos-Coquillat *et al.* reported covalently labeled goat milk EVs using fluorophore sulfo-Cyanine 5, allowing fluorescence imaging of them in inflammatory processes^[71]^. Synthetic metabolic precursors, such as azido-sugar substrates, can be administered and incorporated into the glycoproteins, which can thereby express azido groups capable of click-chemistry reactions with ADIBO dyes^[65]^. An alternative to dyes is Quantum Dots (QDs). Relative to organic dyes, QDs have robust photostability, tuneable excitation/emission, and efficient luminescence, which can aid in longer-exposure imaging. However, broad applications of QDs are still hindered by loading methods^[72]^.

Fluorescent proteins such as CFP and GFP are popular reporter proteins that can emit fluorescent signals at specific wavelengths of excitation light. Fusion proteins are produced by conjugation of fluorescent proteins on the surface or interior of the EV via genetic modification of the parent cell for the latter. Verweij *et al.* pioneered conducting EV research in a zebrafish model, establishing it as a model for observing EV release, transfer and functionality among different organs that were genetically modified to transiently express recombinant pHluorin-CD63 proteins^[73]^. To the best of our knowledge, this is the first *in vivo* tracking of endogenous EVs. *In vivo* fluorescence imaging enables visualization of biology in its complete and native physiological state but does possess inherent technical difficulties. Fluorescence imaging has limited tissue penetration with lower resolution than other modalities, often necessitating animal sacrifice during which the dyes and proteins, which are highly susceptible to photobleaching, are exposed to light. To the best of our knowledge, no 3D optical imaging has been conducted to precisely determine the spatial distribution of EVs in living animals. The lack of 3D imaging ability appears as a drawback for using optical imaging methods to track EVs in small animals, not to mention large *animals* and human subjects. Secondly, the *in vivo* environment is complex, and therefore the imaging probe or contrast agent requires long-term biological stability at the targeted site and the production of high imaging contrast at the intended site^[74]^. Fluorescent dyes have been verified to offer stable signals for EV imaging, albeit commercial dyes like PKH have an *in vivo* half-life of over 100 days, which is significantly more than that of EVs, which have a circulating half-life of 2-30 min with clearance occurring at most in 6 hours, and PKH can diffuse to neighboring cells^[75]^. This persistence and sporadic aggregation/micelle formation has notoriously led to erroneous longitudinal biodistribution analyses. The use of fluorescent proteins also results in a low yield of EVs, as not all the vesicles can successfully express the recombinant variant^[74]^.

##### Bioluminescence imaging

**Figure 5.**
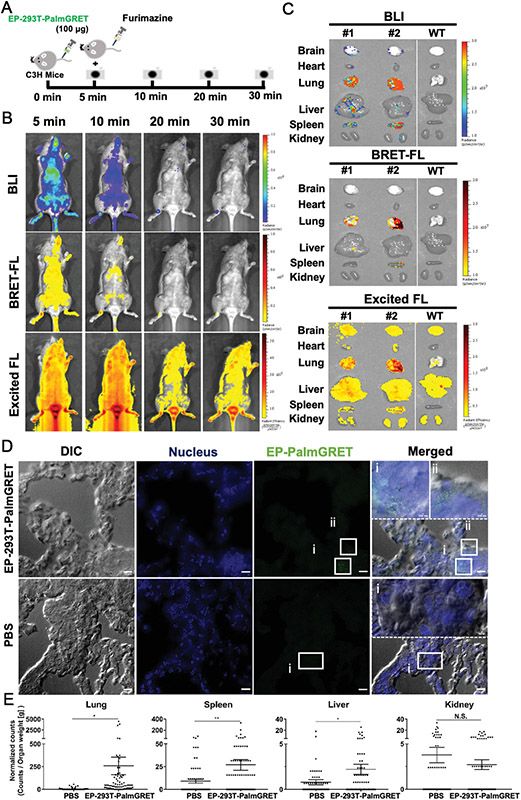
PalmGRET enables multimodal, multiresolution imaging and analysis of extracellular particles (EPs)’ distribution in organs. (A) Schematic of *in vivo* time-lapsed imaging of EPs. Immunocompetent C3H mice were administered with EP-293T-PalmGRET (100 μg) into the tail vein, followed by Fz injection for *in vivo* imaging at 5, 10, 20, and 30 min post-EP administration. (B) Bolus IV-injected EP-293T-PalmGRET (100 μg) was visualized under BLI and BRET-FL (GFP using BRET) channels from 5 to 20 min post-administration. The majority of the EP signals were detected in the lungs and spleen. By contrast, imaging under epi-illumination resulted in mostly scattered, non-EP-specific signals. (C) Ex vivo imaging of organs harvested at 30 min post-injection of EP-293T-PalmGRET (100 μg) (Mice No. 1 and No. 2) and WT subjects. EP-293T-PalmGRET signals were readily detected in the lungs, liver, and spleen. (D) Super-resolution radial fluctuations (SRRF) nanoscopy of lung sections at 30 min post-injection of EP-293T-PalmGRET (100 μg) or PBS (control). Enlarged images (dashed boxes) of boxed regions I.ii) reveal injected EPs in lung tissues. The nuclei were stained by DAPI, and EP-293T-PalmGRET was immunoprobed by anti-GFP antibody followed by AlexaFluor 568-secondary antibody to minimize the background signal. Bar, 10 μm; in enlarged images, 500 nm. (E) Quantification of EP signals by SRRF imaging demonstrated a significant increase in EP counts in the lungs followed by the spleen and liver at 30 min post-EP injection. The kidneys showed no significant increase in EP counts. 293T-PalmGRET signals were quantified by ImageJ from seventy images of tissue sections of mice injected with EP-293T-PalmGRET or PBS. The EP counts were normalized against organ weight. N.S., *P* > 0.05; *P < 0.05; **P < 0.01 with two-tailed Studen’s *t*-test. Reprinted with permission from^[80]^.

Bioluminescence imaging (BLI) is another commonly used optical imaging technique that differs from fluorescent imaging as it does not require an excitation light source. BLI grants real-time visualization of the biodistribution of EVs with a high signal-to-noise ratio, deep tissue penetration, and high specificity. Among currently developed BLI reporter systems, luciferases are the most common class, and bioluminescence occurs when luciferases catalyze luciferin substrates to generate a transient excited complex that releases photons as it reverts to its ground state. While firefly luciferase (Fluc) is the most predominant system being used in preclinical studies, it is not suitable for *in vivo* tracking of EVs because the reaction requires several intracellular co-factors including ATP. ATP-independent marine luciferases such as Gaussia (GLuc), Renilla (RLuc), and Metridia (MLuc), therefore, were explored^[76]^. Takahashi *et al.* reported the first study in which plasmids were constructed to express a recombinant protein consisting of GLuc and a condensed lactadherin (Gluc-LA). Gluc is found to be 1,000-fold more sensitive than Rluc and Fluc^[77]^. By BLI, they studied the biodistribution of EVs derived from Gluc-transfected B16-BL6 murine melanoma cells and observed EVs had an extremely short half-life (2 min), with the first migration occurring to the liver, followed by the lungs. In later studies, more sensitive BLI systems were developed by synthetic biology. Currently, Nanoluciferase (NanoLuc), which uses furimazine as its substrate, has been successfully developed with high sensitivity and a long *in vivo* half-life (> 2 h)^[78]^. With recent successes on substrates that are more suitable for *in vivo* imaging, i.e., fluorofurimazine (FFz)^[79]^, Nanoluc has been adapted in EV imaging in mouse models in the last several years. For example, Wu *et al.* have reported a multimodal, multiresolution imaging and analysis of EVs in mice using both BLI and bioluminescence resonance energy transfer (BRET)- based fluorescence imaging (FL) [Figure 5]^[80]^. Hikita *et al.* have also developed genetically modified HT29 colorectal adenocarcinoma cells to express Nanoluc at the transmembrane protein CD63 which is abundant in EVs. The biodistribution of EVs released from an implanted chamber ring was monitored by bioluminescence and uptake in the stomach was shown to be preferable^[81]^.

Our group transfected cells with palmGRET, a palmitoylated EGFP-Nanoluciferase fusion protein, to prepare EVs that are imageable. Using BLI, the whole-body biodistribution of EVs, administered either intranasally or intravenously, was studied in mice. NanoLuc also allowed assessing the EV concentration in plasma and cerebrospinal fluids in pig-tailed macaques. The study showed that palmGRET facilitates highly sensitive detection and can even be employed in larger animals for pharmacokinetic research. The results showed that EVs circulate longer in the primate as compared to the murine model, with CNS penetration being low in both sets. The results also implied that EVs are retained *in vivo* in a demand-pull manner rather than a supply-push form^[19]^.

This imaging technique can reliantly provide a whole organism image for analyzing EV biodistribution and the physiological processes involved. Although more specific than fluorescence imaging, there is a requisite to inject substrates that have relatively short half-lives (~5 minutes) prior to imaging session, hampering the technology’s application. Finally, as an optical imaging method, BLI also possesses constraints in resolution and tissue penetration depth.

#### Nuclear imaging

Nuclear imaging, including single-photon emission tomography (SPECT) and positron emission tomography (PET), is an imaging modality that is widely used in the clinic. Image probes in the form of radionuclides are detected at concentrations in the pico- to nanomolar ranges using special cameras and confer the advantages of high sensitivity and limitless depth, enabling them to be particularly suitable for non-invasive tracking of EVs *in vivo*.

Radiolabeling of EVs is typically carried out by tagging the surface proteins or lipophilic membrane of the vesicle. This strategy can be achieved via lipophilic diffusion, genetic modification, chelator mediated conjugation or direct incorporation into membrane proteins by targeting their amine groups. Alternatively, the radiolabel can be entrapped in the intra-vesicular space. It relies on the presence of glutathione, which can modify complexes from lipophilic to hydrophilic states to get trapped in the aqueous core, or on an ionophore-chelator binding method that exploits ionophore ligands, such as 8-hydroxyquinoline(oxine) and tropolone, which form a neutral, metastable amalgam with the radioligands, allowing them to transport across the phospholipid layer and bind to internal proteins^[82]^.

##### SPECT

SPECT detects the emission of γ photons released during the decay of radionuclides injected. The gamma rays are filtered by collimators and captured by a rotating camera which relays the data that undergoes processing to obtain 3D images that accurately depict biodistribution of the tracer. Morisha *et al.* were the first to radiolabel EVs with a yield of around 80% by substituting lactadherin with streptavidin which has a strong binding affinity for biotin^[57]^. ^125^I was conjugated to biotin and utilized for biodistribution analyses of B16-BL6, which over the course of 4 h showed significantly different profiles for free tracer relative to the EV conjugated variant, with the latter being concentrated in the liver, lung and spleen. Thyroid uptake was negligible, which is surprising as the organ has Na^+^/I^−^ symporters. ^111^In-tropolone and ^111^In-oxinate allow intraluminal radiolabeling, with the former being more stable and resistant to transchelation with the caveat that it is less efficient for intraluminal radiolabeling. Later, Faruqu *et al.* utilized ^111^In-tropolone to achieve a labeling yield of 4.73% ± 0.39% for EVs^[69]^. Regular purification methods involve size exclusion columns such as Sepharose which results in a loss of 50% of the original stock of EVs which was verified in this study as well. The authors compared the biodistribution of surface-labeled and intraluminally labeled EVs and established that the strategies can affect pharmacokinetics of the vesicles but also highlighted that biodistribution does not get impacted by the immunocompetency of an organism except in relative intensity of tumor uptake due to diminished populations of circulating macrophages in immunocompromised mice^[69]^. With a short half-life of 6 h, ^99m^Tc is a preferred radioactive ligand due to its widespread availability and lower costs. A recent example of its use was by Giraud *et al.*, wherein the group labeled endothelial EVs with ^99m^Tc-Annexin-V-128, which binds to phosphatidylserine molecules present in the EV membrane to track its biodistribution in a mouse model of peripheral ischemia. In a week, EVs promoted a significantly earlier and higher vascular recovery and a positive correlation with the concentration of EVs addressed to the site was observed after 28 days. Despite being the most widely used radionuclide, the short half-life of ^99^mTc only allows imaging for up to 24 h post administration, which is not suitable for long-term *in vivo* tracking of EVs^[83]^. ^131^I is another convenient ligand that can be used to conveniently radiolabel EVs derived from various cell lines with a half-life of 8 days^[84]^.

##### PET

Although more expensive, PET offers higher spatiotemporal resolution than SPECT. PET requires a circular ring of detectors around the subject for coincidence detection, i.e., the detection of the annihilation event of a positron emitted from the decaying radioisotope and an electron in the surrounding tissue that produces a pair of gamma photons traveling at 180° away from each other^[85]^. Given the ultra-high sensitivity, accurate quantification, and excellent clinical translatability, an increasing trend towards employing PET for EV research has been observed in the past years. Shi *et al.* used PET to assess the biodistribution of PEGylated EVs derived with 4T1 breast cancer cells conjugated with amine-reactive ^64^Cu-NOTA, which had a very high yield for labeling (~95%). The group observed the favorable characteristic bestowed by the PEG coating, namely enhanced tumor uptake and minimal liver uptake 24 hours post-injection^[85]^.

**Figure 6.**
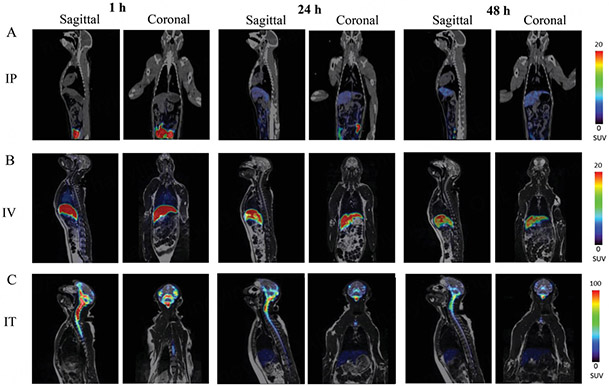
Representative PET/MRI images of ^64^Cu-labeled EVs in NHP. Macrophage derived EVs were labeled with ^64^Cu and injected into rhesus macaque monkey (0.3 mCi/1012 particle mL-1, N = 4) via three administration routes; (A) IP, (B) IV, and (C) IT. Animals were imaged by PET/MRI over 24 h-time period after the injection. Sagittal and coronal representative images of animals suggest that 64Cu-EVs accumulate in the brain of IT injected animals at greater extent than those with IP, and IV injections. Reprinted with permission from^[86]^.

One of the unbeatable advantages of PET imaging is the ability to translate to large animal and human studies. There are two recent studies in which PET has been successfully used to track the biodistribution of EVs in nonhuman primates. In the report by Haney *et al.*, the biodistribution of immunocyte-based carriers, peripheral blood mononuclear cells (PBMCs), and monocyte-derived EVs are investigated in adult rhesus macaques using longitudinal ^64^Cu-PET/MRI imaging [Figure 6]^[86]^. The authors concluded that a significant amount of EVs can reach the brain, with the retention highly related to the route of administration, i.e., intraperitoneal < intravenous < intratumoral. Interestingly, intratumoral injection favors the brain retention of PBMCs compared to monocyte-derived EVs, while intraperitoneal and intravenous routes showed totally opposite effects. In another study, the pharmacokinetics and biodistribution of ^89^Zr-labeled engineered extracellular vesicles were studied in both rodent and nonhuman primate subjects^[87]^. The authors compared different CSF administration routes, including intrathecal (IT), intracisterna magna (ICM), and intracerebroventricular (ICV). Heterogenous distribution patterns were observed among different routes, with IT administration tending to result in meningeal distribution along the neuraxis to the base of the skull, while ICM and ICV dosing resulted in meningeal distribution around the skull and to the cervical and thoracic spinal column. These two studies clearly showed the feasibility of translating the PET imaging developed in preclinical models to large animals and potentially human subjects. Moreover, the refined findings of biodistribution in nonhuman primates are essential for designing optimal administration routes to boost the efficacy of EV therapy, implying the great clinical potential and large market values of theranostic EVs.

Restrictions to PET’s ubiquitous use lie in its expenses related to the requisite of cyclotrons for the production of radionuclides. A trained researcher is required for the operation of the scanner and for handling the radioactive molecules, which from the name itself implies that it is hazardous and may not be widely permissible for clinical translation of EV research. Radiolabeling also has a few drawbacks. Intraluminal radiolabeling approaches avoid interaction with serum proteins but do not display a high labeling yield, and nor do we have sufficient data to support the exact mechanism or component the radiolabel binds to in the aqueous core^[88]^. Complications from this arise in the interpretation of processed data, especially those collected at later time points, as EVs are quite unstable *in vivo* and disintegrate much faster than the radiolabels and lead to biasing in reported data as some labels aggregate in the same organs. In many studies, substantial accumulation in the bladder was indicated, which would only occur if EVs could clear glomerular filtration in kidneys implying that the vesicles would have to be < 8 nm due to quick disintegration in serum *in vivo* with radiotracers attached to EV components being cleared^[89]^. Most studies apply nucleic acids and proteins as targets for biomarkers and have disregarded the glycosylation profile, which occupies a significantly higher volume than proteins and is involved in hepatic clearance. Royo *et al.* radiolabeled the proteins of EVs with a depleted sialic acid content with the oxidized version of ^124^I. PET scans revealed that liver accumulation occurred at a mere 30 s after I.V. injection and joint administration led to projected lymphatic drainage^[90]^.

#### Computed tomography

Ultra-small gold nanoparticles have been explored as a tracer for CT imaging of EVs. Gold nanoparticles are biocompatible, nontoxic, and can have highly tuneable functional groups and coatings with strong X-ray attenuation. Perets *et al.* intranasally administered mesenchymal stem cell- derived EVs labeled topically with glucose-coated gold nanoparticles (GoldenExos), which enabled EV uptake via an energy-dependent translocation carried out by GLUT-1 transporters^[91]^. The results showed that EVs were preferentially taken up in the brain in a physiologically inflamed state as that of in ischemic stroke, Alzheimer’s, Parkinson’s and autism and could be detected even 96 hours post-delivery. GoldenExos had high homing characteristics toward specific neuropathology regions, especially in neurons, possibly due to inherent ligands derived from the EV releasing cells^[91]^. Lara *et al.* incubated different cell types with folic acid coated nanoparticles and exploited the endocytic pathway for consequently secreting EVs with encapsulated nanoparticles, without altering the natural tropism of the obtained vesicles^[92]^. CT scans obtained confirmed that oncological tissues preferentially take up EVs originating from a similar source. The findings are of importance and can potentially lead to more effective drug delivery strategies for metastatic tumors^[92]^.

#### Photoacoustic imaging

Photoacoustic imaging (PAI) is an emerging non-invasive imaging modality accomplished by delivering pulsed non-ionizing laser beams into tissues whose energy will be partially absorbed, resulting in a transient thermoelastic expansion effect that emits ultrasonic waves in the range of MHz. Advantages of photoacoustic imaging include a high spatial resolution, high contrast and deep penetration depth. Photosensitizer chlorin e6, an oxaliplatin precursor, has also been loaded via sonication in tumor-derived EVs for PAI detection of EV uptake in tumors and stimulating an immunomodulatory effect *in vivo*^[93]^. Gold nanostars, which have exceptional optical properties, have been demonstrated in detecting tumor cells transplanted in mice^[94]^. Piao *et al.* used PAI to monitor tumor growth and axillary lymph node metastasis^[95]^. Graphene quantum dot nanozyme (GQDzyme), with its intrinsic peroxidase activity, can effectively convert ABTS (3-ethylbenzothiazo-line-6-sulfonic acid) to its oxidation state, ABTS+, in the presence of H_2_O_2_. The latter has a strong near-infrared (NIR) absorption capacity for PAI^[96]^. In another recent study, PAI was used to track the delivery of indocyanine green (ICG)-labeled, therapeutic miRNAs-loaded EVs to experimental glioma by means of intranasal administration^[97]^. The delivered miRNAs sensitized the tumor cells to temozolomide, leading to significant tumor regression, and improved the overall survival of mice. It should be noted that ICG is an FDA-approved agent, making the system highly translatable.

#### Magnetic particle imaging

MPI is a relatively new imaging modality that only became commercially available in 2014. MPI instrumentation involves creating a magnetic field-free region (FFR) via a unique geometry of magnets positioned in the device, and the FFR is scanned 3 dimensionally to map the location and quantity of magnetic nanoparticles. MPI is an imaging modality with minimal background signals (hot spot imaging), higher signal- to-noise ratios, and (linear) quantification for detecting magnetic nanoparticles without the limitation of tissue depth. It can be used in diverse applications, from diagnostics to therapeutic applications in the form of magnetic hyperthermia without the use of ionizing radiation^[98]^. SPIONs have surfaced as the most popular nanoparticles for the modality with many commercial or FDA-approved formulations such as Feraheme and Vivotrax, due to their superparamagnetic properties, which permit high-order harmonics of excitation frequencies under an oscillating field for quantitative localization analyses. Moreover, SPIONs are inherently biocompatible with easily tunable size and modification of surface functionalization. The core size of SPIONs is crucial to control with ideal particles lying between 10-100 nm as it strongly affects the amplitude of the MPI signal, and a core size of 20 nm is generally considered to be optimal. The first and only published study to date on using MPI to track EVs was conducted by Jung *et al.*, where the group confirmed liver and hypoxic tumor uptake of MDA-MB-231 breast cancer cell-derived EVs loaded with a poly ADP ribose polymerase (PARP) inhibitor and SPIONs 1-hour post-injection in MDI-MB-231 xenograft mice and also displayed retarding tumor growth^[99]^. As MPI is an emerging non-invasive imaging technique, it has not been scaled up yet for clinical trials, nor is there enough published work yet for EV tracking. However, there are a few ongoing studies.

#### Magnetic resonance imaging

MRI is routinely used clinically for its high spatial and soft tissue resolutions. However, MRI has not yet been extensively explored for EV imaging, likely due to the relatively higher requirement of the concentration of MRI agents to be attached to EVs in order to achieve sufficient MRI signal. Currently reported contrasts for labeling of EVs for *in vivo* tracking include SPIONs, gadolinium, manganese nanoparticles, and hyperpolarized chemical exchange saturation transfer (CEST) agents. The main disadvantage of MRI relative to other imaging modalities is its requirement for a supply of higher concentrations of contrast agents for sensitive detection.

As one of the most widely used T2/T2* MRI contrast agents, SPIONs are the first type of MRI agent applied to EV detection. In 2015, Hu *et al.* first demonstrated the *in vivo* MRI detection of SPION loaded melanoma-derived EVs in popliteal lymph nodes after injection of 50 μg EVs (protein content, 27.45 μg SPION) to one foot pad of the mice^[100]^. The SPIONs were loaded to EVs using electroporation that was optimized in detail, as reported in an earlier study^[101]^. The advantage is the ease of preparation and high loading efficiency. However, it requires a sophisticated ultracentrifuge procedure to remove unencapsulated SPIONs from EVs, via which obtaining a high purifying efficiency is challenging. In a later study by Busato^[102]^, the parental cells were first incubated with adipose stem cells (ASCs) to include SPIONs, and the secreted ASC-EVs were subsequently collected, in which a portion of EVs contained SPIONs. This strategy has been used in a few studies and gained some success^[103,104]^. Recently, this method was utilized for the MRI detection of the binding of receptor binding domain (RBD) of SARS-CoV-2 to ACE2 receptors using SPION-loaded genetically engineered EVs that display the receptor binding domain (RBD) of SARS-CoV-2 on their surface as coronavirus mimetics (EVsRBD)^[105]^. The inherent drawback of this approach is the low labeling efficiency. In fact, a recent paper reported controversial results in which no SPIO-containing EVs were obtained after incubating human MSCs with Molday (size = 35 nm) at a concentration of 20 μg/ml for 16 hours^[106]^. In addition, incubating cells with SPIONs can result in unwanted changes in the cells. In a recent study, RNA-seq was used to compare EVs derived from native MSCs and SPION-labeled MSCs, revealing a significant change in various miRNA levels, although the authors claim the effect was mainly to increase therapeutic molecules^[107]^. Thirdly, SPIONs can be conjugated with antibodies^[108]^ or amines^[109]^ on the surface of EVs.

**Figure 7.**
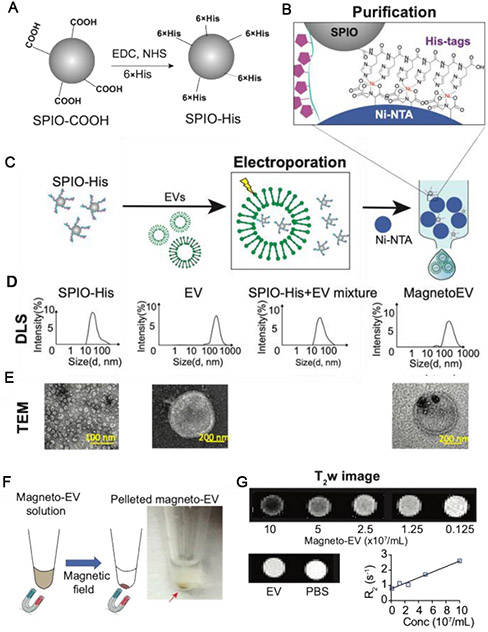
Preparation of magneto-EV and characterization of purified magneto-EVs. (A) Schematic illustration of the preparation of SPIO his-tag (SPIO-His), by conjugating hexahistidine (6 × His-tag) polypeptide to the carboxyl groups of SPIO particles using EDC (1-ethyl-3-(3-dimethylaminopropyl) carbodiimide), and NHS (sulfo-N-hydroxysuccinimide) chemistry. (B) As a result of the high affinity between the His-peptide and nickel ion, the SPIO-His particles bind to Ni^2+^ immobilized on beads (e.g., Ni-NTA resins) for further purification. (C) Schematic illustration of the encapsulation of SPIO-His into EVs by electroporation and subsequent purification by removing unencapsulated SPIO-His from the elute using Ni-NTA affinity chromatography. (D) Size distribution as measured by dynamic light scattering (DLS) for SPIO-His, EVs, SPIO-His/magneto-EV/EV mixtures after electroporation, and the final purified elute, respectively. (E) TEM images of EV, SPIO-His and EV-SPIO, respectively. (F) Concentrating magneto-EVs using a magnet. Eluted magneto-EV solution was placed on a magnet overnight to pellet magneto-EVs. The photograph shows the pelleted magneto-EV at the bottom of a microcentrifuge tube. (G) T2-weighted (T2w) images of magneto-EVs at different concentrations, unlabeled EVs, and PBS. Mean R2 values of magneto-EVs are plotted with respect to their concentration, from which the r2 (relaxivity) was estimated. Reprinted with permission from^[18]^.

**Figure 8.**
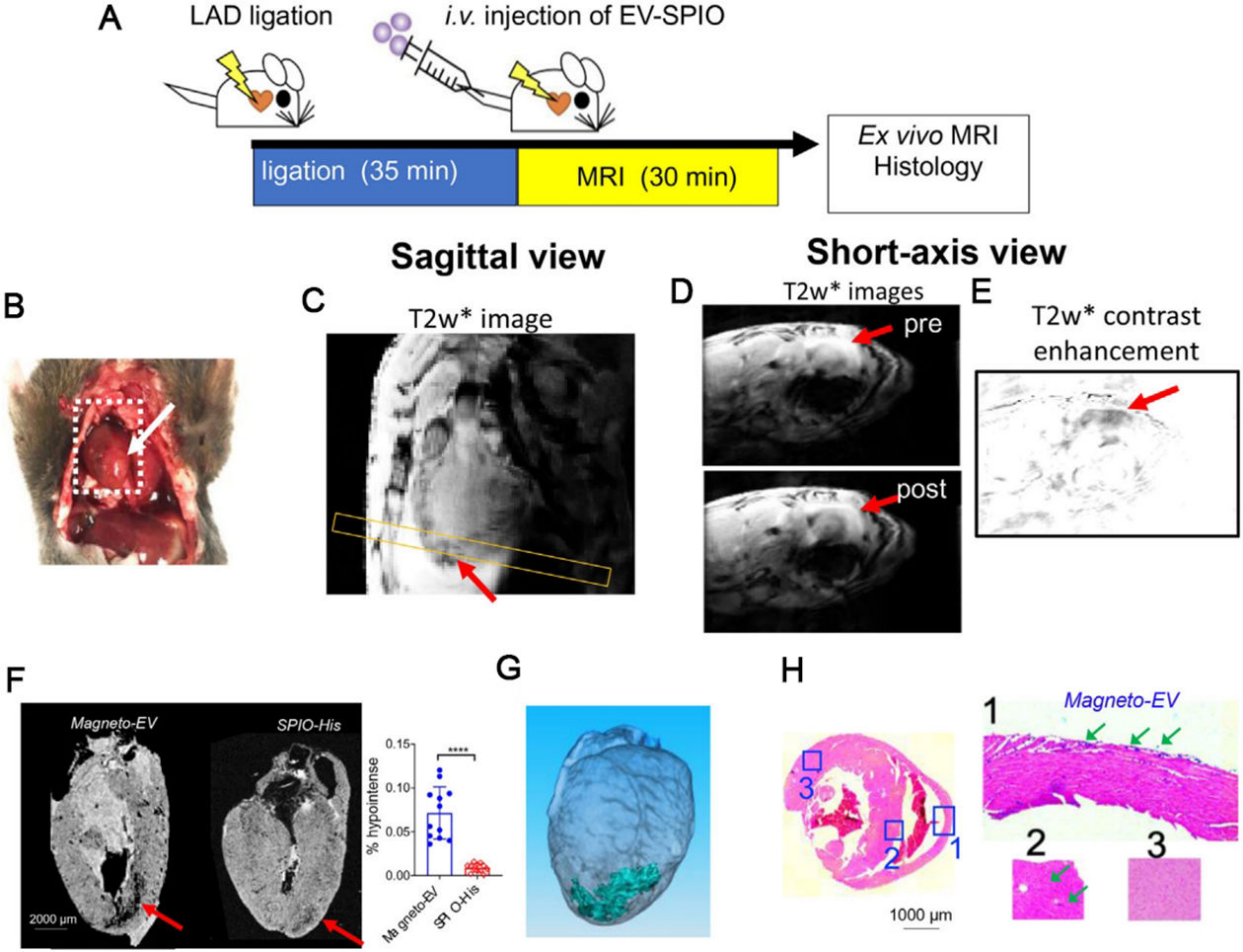
MRI tracking of magneto-EV accumulation in the IR heart. (A) Schematic illustration of the experimental myocardial infarction mouse model and MRI acquisition. (B) Macrophotograph of the heart with the IR region (arrow). (C) Sagittal *in vivo* MR images of the heart. Yellow box indicates the slice position of the short-axis view. Short-axis pre- and post-injection *in vivo* T2*w images (D) and enhancement maps, defined as T2*w = T2*w (post)-T2*w (pre) (E) showing hypointense areas in the injured region around the apex of the heart. (F) Ex vivo heart MR image showing higher accumulation of magneto-EVs (red arrow) in injury region than that of SPIO-His. The measured percentages of hypointense area in the myocardium of mice that received magneto-EVs or SPIO-His are shown on the right. A total of 12 ex vivo MRI image slices were analyzed for three mice in each group. ****P < 0.0001, unpaired two-tailed Student’s t-test. (G) 3D reconstruction showing the distribution of magneto-EVs in the hearts. (H) Prussian blue staining of the injured heart (Left: whole heart of axial view; Right: zoom-in of sections 1-3). Reprinted with permission from^[18]^.

In our recent studies, we have developed surface-functionalized SPIONs to facilitate the preparation of magnetically labeled EVs^[18]^. As illustrated in Figure 7, we modified the surface of commercial SPIONs with 6-histidine tags, which allows them can be efficiently separated from EVs by Ni-NTA columns, making the post-loading purification procedure simple, less equipment-intensive, and much faster (~min). The prepared EVs have an estimated MRI detection limit of approximately 8.76 × 10^7^ EVs per ml, which is sufficient for many therapeutic applications. Our approach has allowed high-resolution MRI detection of the uptake of intravenously injected iPSC-EVs (a single dose of 2 × 10^9^ EVs) in the injured heart [Figure 8] as well as several other animal models and have shown to provide protection in injuries^[18]^.

**Figure 9.**
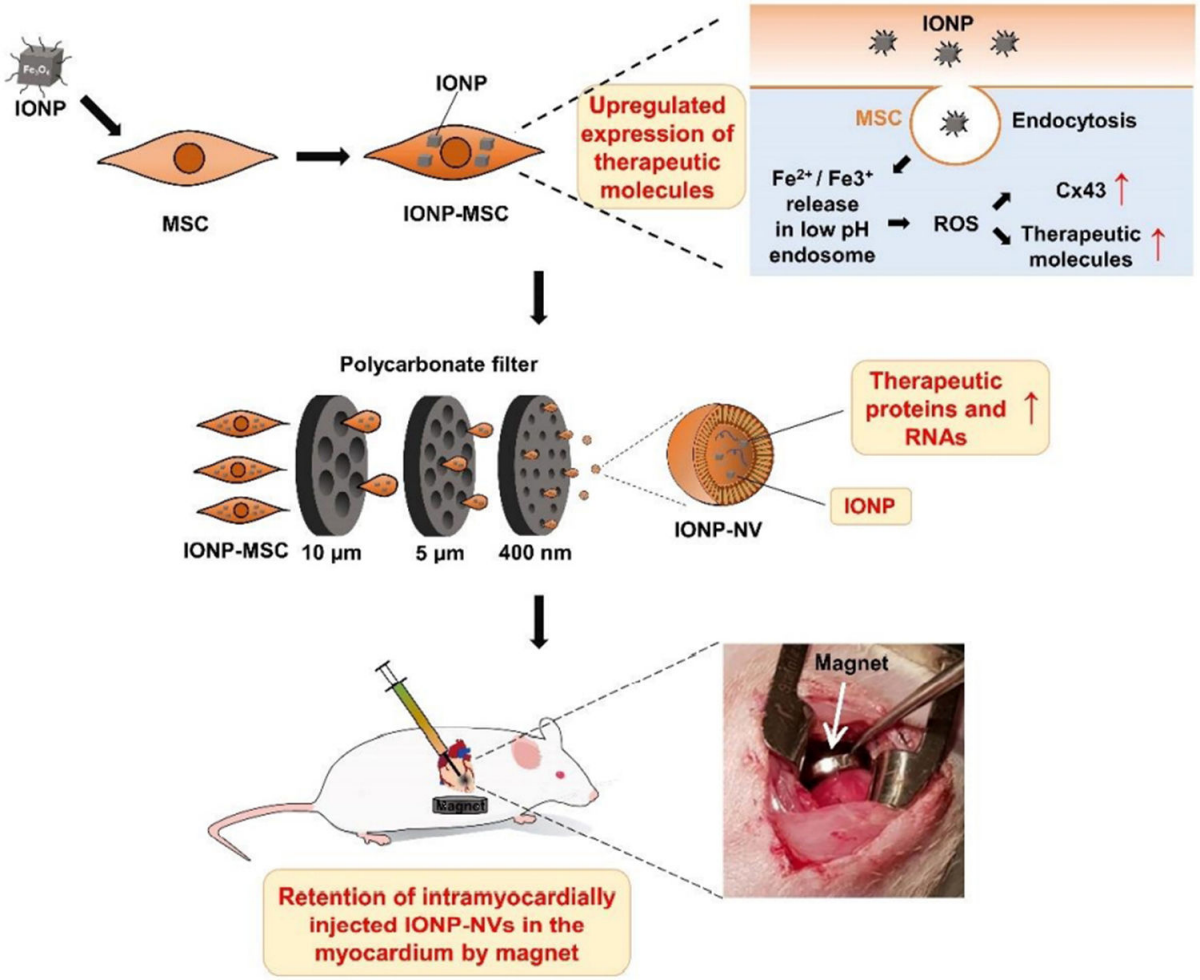
Schematic diagrams for the preparation and intramyocardial injection of IONP-NVs for cardiac repair. MSCs modified through the internalization of IONPs exhibited higher expression of therapeutic biomolecules. IONP-NVs were generated from IONP-MSCs by serial extrusion and contained contents similar to those of IONP-MSCs. Magnetic guidance following intramyocardial injection of IONP-NVs can improve cardiac retention of IONP-NVs. Reprinted with permission from^[107]^.

**Figure 10.**
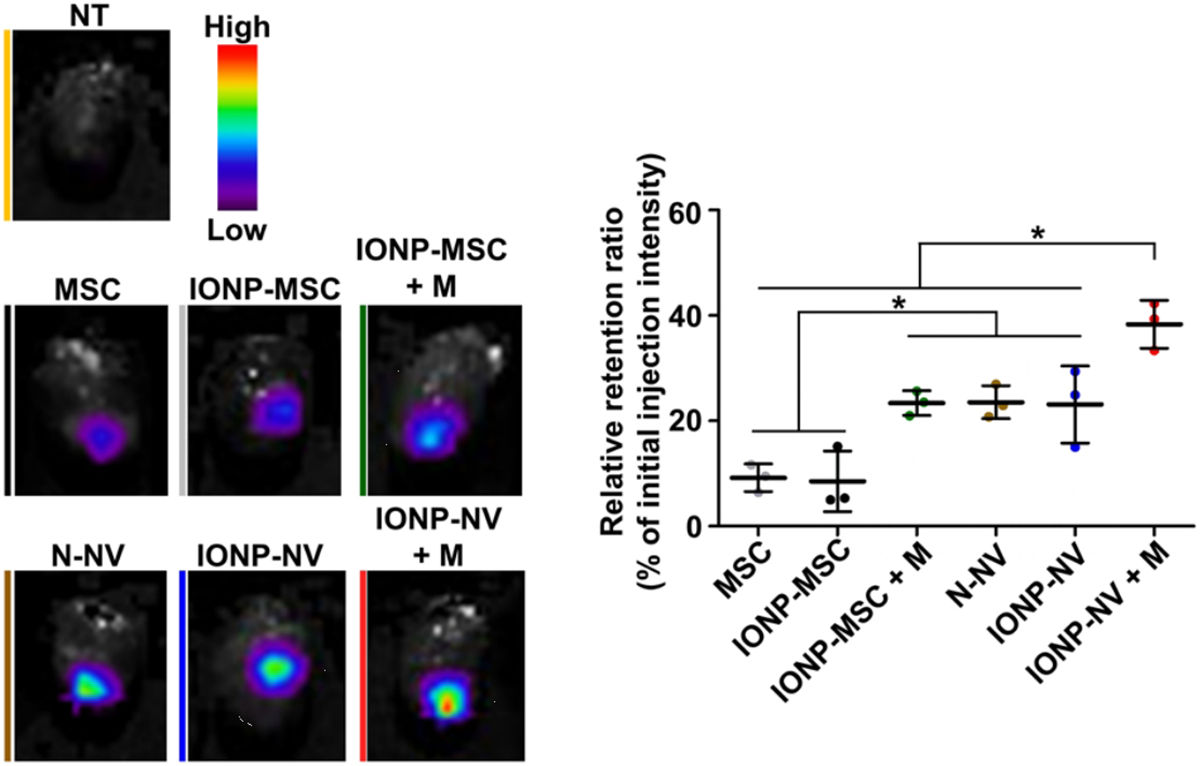
Representative ex vivo fluorescent imaging of infarcted hearts and the quantitative data 24 h after the injection of VivoTrack 680-labeled MSCs, IONP-MSCs, N-NVs, and IONP-NVs (*n* = 3 animals per group). VivoTrack 680 labeled the membranes of cells and NVs. *P < 0.05, using one-way ANOVA, followed by post hoc Bonferroni test. Reprinted with permission from^[107]^.

In addition to imaging, the magnetic properties of SPION can be utilized for magnetic targeting. For instance, Lee, at al demonstrated the use of magnet to increase the retention of EVs in the heart [Figure 9]^[107]^. Their data showed that SPION-loaded EV+ magnetic guidance group exhibited the highest retention in the infarcted heart (Figure 10). The magnetic guidance was achieved simply using a neodymium magnet that was implanted into the muscle above the heart for 24 hours. Through immunohistochemistry, it was shown that the magnetic vesicles could effectively induce early transition of the inflammatory phase to the reparative phase by facilitating the rapid polarization of M1 macrophages into M2 macrophages enabling cardiac repair after a day from injection time. It also improved blood vessel density and decreased the fibrosis and scar area in treatment groups. As a proof-of-concept study, it clearly demonstrates the feasibility of forging multi-functional theranostic EVs simply by the SPION that are either conjugated to or encapsulated in EVs.

While the current efforts to develop MRI-visible EVs mainly focus on SPIO-based T2 and T2* imaging, other MRI contrast mechanisms may also be possible to be used to track EVs. In this context, Gd-based T1w contrast agents have been explored. Abello *et al.* first reported a direct post-insertion method to label EVs using gadolinium lipid (Exo-GdL)^[110]^. They then used MRI to study the biodistribution of labeled human umbilical cord mesenchymal stromal cells (HUC-MSCs) and found the tendency to accumulate within human or mouse osteosarcoma cells both *in vitro* and *in vivo*. Besides MSC-EVs, the same group also constructed gadolinium infused hybrid macrophage-derived EVs via membrane fusion^[111]^. Zhao *et al.* conjugated Mn^2+^ on the surface of quantum dots to construct a multimodal ultrasmall Mn-magneto-functionalized Ag_2_Se quantum dots (Ag_2_Se@Mn QDs) that integrated both near-infrared (NIR) fluorescence and MRI capabilities for *in vivo* high-resolution dual-mode tracking of microvesicles (MVs)^[112]^. However, the MRI contrast enhancement is only marginal compared to NIR signal.

Another type of MRI contrast that has potential for EV imaging is a so-called Chemical Exchange Saturation Transfer (CEST), which was first developed in 2000. CEST contrast is generated through the exchange of magnetism carried by exchangeable protons to the surrounding water pool. In a typical CEST acquisition, saturation pulses are applied to irradiate and saturate the exchangeable protons, which subsequently cause the attenuation in water signal. Diamagnetic solutes such as glucose, amines and lipids have been used to generate a CEST signal and map pH and changes in the microenvironment of tissues which is particularly valuable in pathophysiological states, and therefore, EVs can theoretically be imaged using their inherent composition^[113]^. To date, only one ^129^Xe-based hyperpolarized CEST (Hyper-CEST) agent has been reported to label and image EVs^[51]^.

## CONCLUSION AND OUTLOOK

In this review paper, we have provided a brief review of the current progress of EV theranostic research, focusing on the imaging modalities used for tracking them *in vivo* [Table 1] and their therapeutic potential. Encouraging results have been obtained using various labeling strategies and imaging techniques that are applied to a wide spectrum of EVs. As implied by these studies, it is clear that EVs can be fabricated toward a next-generation theragnostic platform. Among all the possible research areas, we envision the following two directions may have high potential and deserve more attention.

First, multimodal imaging can be a powerful means to uncover the behavior of EVs. Many of the abovementioned imaging modalities are complementary and can be used combinedly. Zhao *et al.* synthesized Ag_2_Se Quantum Dots that are capable of both MRI and near-infrared (NIR) fluorescence bimodal imaging for supporting results of EVs biodistribution in murine tumor models^[112]^.

Secondly, hybrids of EVs and synthetic membrane nanoparticles such as liposomes can be used to prepare more robust theranostic systems. In the last few decades, synthetic nanoparticles such as liposomes, dendrimers and self-assembled peptides have been developed for nanomedicine applications but have limited success. EVs have high biocompatibility and specific tropism depending on the type and status of the secreting cells. EVs can escape phagocytosis as they possess high levels of the tetraspanin CD47 that results in immune evasion via the CD47- SIRPα receptor interaction^[114]^. Moreover, endogenous cellular components include molecules that aid in adhering to cellular membranes and facilitate traversing target cells’ phospholipid membranes. As studies involving native extracellular vesicles for diagnostic and therapeutic applications are challenged by scale-up, purification, membrane integrity loss during loading and freedom of surface modification, the production of hybrid particles by the fusion of liposomes and EVs is highly sought after and being researched.

It should be noted that the development of EV therapeutics requires large-scale cell culture that is still technically challenging, although stirred- tank or fixed-bed bioreactors are being investigated. The process in which engineered EVs are formed by physical force or chemical stimuli may impact membrane topology^[115]^. The complete characterization of EVs warrants future clinically ready forms of theranostic EVs.

## Figures and Tables

**Table 1.** Characteristics of imaging modalities presented along with a summary of their advantages and disadvantages

Imaging modality	Examples of agents	Spatial resolution	Limit of depth of imaging	Sensitivity of detection	Duration of signal	Advantages	Disadvantages
Fluorescence imaging	Carbocyanine dyes Lipophilic dyes Fluorescent protein reporters Quantum dots	20 μm (IVIS systems)	1-10 cm	10^−9^-10^−11^ M	12-24 h	Convenient to use Low cost Labels are small and may not affect EVs Imaging artifacts are rare Quick scan time	Photobleaching will affect detection Genetic modifications may disrupt biofunctionality Restricted to small animal models Tissue light attenuation constraints deep tissue imaging Lack of 3D imaging ability Anatomical information needs to be provided by MRI or CT
Bioluminescent Imaging/BRET	Firefly/Gaussia Luciferases palmGRET	1-5 mm	1-5 cm	10^−9^-10^−11^ M	Days - substrate needs to be provided	Labels are small and may not affect EVs Imaging artifacts are rare Quick scan time High SNR	Requires genetic modification and substrate administration which would not be FDA-approved Anatomical image would be required via MRI or CT Difficult to control substrate localization at target
SPECT	^99^mTc ^111^In ^131^I, ^125^I	1-2 mm	None	10^−10^-10^−11^ M	6-48 h	Label shows up as hot spots with rare artifacts	Long scan times Uses radioactive tracers Low spatial resolution Tracers tend to leach from EV surface
PET	^64^Cu ^89^Zr ^18^F	1-2 mm	None	10^−11^-10^−12^ M	1-24 h	Quantitative whole body imaging	Long scan times Radioactive tracers need to be administered Uses radioactive tracers Radiodecay leads to short half-lives Expensive and requires specialized instrumentation May require a radiochemist
CT	Gold nanoparticles	50-200 μm	None	10^−3^-10^−4^ M	96 h	Long shelf-life of imaging agents Natural tropism of EVs not found to be altered Short scan time	Exposure to X-ray radiation
Photoacoustic Imaging	Chlorin e6 Gold nanostars	15 μm	2-3 cm	10^−6^-10^−7^ M	4-24 h	High sensitivity High spatial resolution Does not require any radiation	High cost of instruments
MPI	SPIONs	1 mm	None	10^−3^-10^−4^ M	2-3 weeks.	No radiation is involved Long shelf life of agents Clinical translatability Whole body quantification	Scanners available for small animals yet so far Requires administration of ferromagnetic materials Size of agent may interfere with EV function Anatomical context needs to be provided by MRI or CT
MRI	Gd chelates SPIONs USPIONs Mn^2+^ Quantum dots	25 μm-100 mm	None	10^−3^-10^−5^ M	2 weeks	Useful for obtaining anatomical images No radiation involved	Unsuitable for patients with metal implants Expensive instrumentation and requires specialized tools that will not get affected by the large magnet used in scans Imaging agents may alter EV functions Gd can cause nephrotoxicity Long scan times depending on pulse sequence Can be noisy during scan
